# Docetaxel-Loaded Poly(3HB-*co*-4HB) Biodegradable Nanoparticles: Impact of Copolymer Composition

**DOI:** 10.3390/nano10112123

**Published:** 2020-10-26

**Authors:** A.F. Faisalina, Fabio Sonvico, Paolo Colombo, A.A. Amirul, H.A. Wahab, Mohamed Isa Abdul Majid

**Affiliations:** 1 Malaysian Institute of Pharmaceuticals and Nutraceuticals (IPharm), National Institute of Biotechnology Malaysia (NIBM), Ministry of Science, Technology and Innovation (MOSTI), Penang 11800, Malaysia; faisalinafisol@gmail.com (A.F.F.); amirul@usm.my (A.A.A.); isa_majid@usm.my (M.I.A.M.); 2 Food and Drug Department, University of Parma, 43124 (PR) Parma, Italy; paolo.colombo@unipr.it; 3 School of Biological Sciences, Universiti Sains Malaysia, Penang 11800, Malaysia

**Keywords:** biodegradable polymers, nanoparticles, cancer chemotherapy, controlled release, drug delivery systems

## Abstract

Polyhydroxyalkanoate (PHA) copolymers show a relatively higher in vivo degradation rate compared to other PHAs, thus, they receive a great deal of attention for a wide range of medical applications. Nanoparticles (NPs) loaded with poorly water-soluble anticancer drug docetaxel (DCX) were produced using poly(3-hydroxybutyrate-*co*-4-hydroxybutyrate), P(3HB-*co*-4HB), copolymers biosynthesised from *Cupriavidus malaysiensis* USMAA1020 isolated from the Malaysian environment. Three copolymers with different molar proportions of 4-hydroxybutirate (4HB) were used: 16% (PHB16), 30% (PHB30) and 70% (PHB70) 4HB-containing P(3HB-*co*-4HB). Blank and DCX-loaded nanoparticles were then characterized for their size and size distribution, surface charge, encapsulation efficiency and drug release. Preformulation studies showed that an optimised formulation could be achieved through the emulsification/solvent evaporation method using PHB70 with the addition of 1.0% PVA, as stabilizer and 0.03% VitE-TPGS, as surfactant. DCX-loaded PHB70 nanoparticles (DCX-PHB70) gave the desired particle size distribution in terms of average particle size around 150 nm and narrow particle size distribution (polydispersity index (PDI) below 0.100). The encapsulation efficiency result showed that at 30% w/w drug-to-polymer ratio: DCX- PHB16 NPs were able to encapsulate up to 42% of DCX; DCX-PHB30 NPs encapsulated up to 46% of DCX and DCX-PHB70 NPs encapsulated up to 50% of DCX within the nanoparticle system. Approximately 60% of DCX was released from the DCX-PHB70 NPs within 7 days for 5%, 10% and 20% of drug-to-polymer ratio while for the 30% and 40% drug-to-polymer ratios, an almost complete drug release (98%) after 7 days of incubation was observed.

## 1. Introduction

In the last decades, remarkable progress has been witnessed in the research and development of biocompatible and biodegradable polymers for drug delivery and in particular for their use in micro/nanoparticle manufacturing [1]. Biodegradable polymers such as poly(glycolide) (PGA), poly(lactic acid) (PLA), poly(lactide-*co*-glycolide) (PLGA), poly(ε-caprolactone) (PCL), poly(3-hydroxybutyrate) (PHB), poly(2-hydroxybutyrate-*co*-3-hydroxyvalerate) (PHBV), gelatine, chitosan (CHI), and alginate (ALG) are among popular polymers being used for this purpose [2,3,4,5]. Another polymer which appears interesting for the preparation of biocompatible and biodegradable nanoparticles is poly(3-hydroxybutyrate-*co*-4-hydroxybutyrate), P(3HB-*co*-4HB) [6,7].

P(3HB-*co*-4HB) is a biodegradable polymer from the polyhydroxyalkanoates (PHA) group that has properties similar to synthetic thermoplastics. This polymer can be tailored by varying its composition to achieve a range of materials with different mechanical and functional characteristics, from hard crystalline plastics to very elastic rubbers [6,8]. The in vivo degradation kinetics of P(3HB-*co*-4HB) are relatively fast compared to other PHAs, and they can be modulated by varying the 4-hydroxybutyrate fraction [9]. Generally, P(3HB-*co*-4HB) is produced by feeding precursor carbon sources such as 4-hydroxybutyric acid, 1,4-butanediol and γ-butyrolactone to diverse wild-type bacteria. In particular, wild-type and recombinant strains of *Cupriavidus necator* and *Delftia acidovorans* are commonly investigated for P(3HB-*co*-4HB) production using various carbon precursors, in addition to *Escherichia coli* which can be genetically modified to produce this copolymer from unrelated carbon sources, such as glucose [10,11]. P(3HB-*co*-4HB) has attracted special interest among PHAs in the biomedical field since the hydrolysis of P(3HB-*co*-4HB) yields 4HB, a natural human metabolite present in blood. P(4HB) is metabolized by depolymerases, lipases as well as esterases [12,13]. The utilization of large doses of this polymer in the medical field has been approved in view of the relatively slow rate conversion of P(4HB) to 4HB and due to the fact that P(4HB) exhibited high tolerance in vivo. This polymer is thus considered as an excellent biomaterial as it adheres to the strict regulations from the Food and Drug Administration (FDA) [6]. As a consequence, P(3HB-*co*-4HB) has been proposed for a wide variety of biomedical applications such as in-tissue engineering [14,15], wound healing [16] and the drug delivery field [17,18]. In particular, PHB applications as drug carriers for anticancer compounds have received significant attention [7,19,20]. However, the proper formulation of the microsphere/microcapsule/nanoparticles of PHB containing the desired drugs remains an aspect of important concern to ensure that the properties of the drug and/or PHB are not affected and that the release speed of the drug is close to its target [21]. Encouraged by this, in this study, we report the utilisation of P(3HB-*co*-4HB) biosynthesized from *Cupriavidus malaysiensis* USMAA1020, isolated from sludge samples obtained from a local lake in Kulim, Malaysia [22] for the preparation of nanoparticles for delivery of the anticancer drug docetaxel. 

Docetaxel (DCX) is a second-generation taxane semi-synthetically derived from a precursor extracted from the needles of the European yew tree, *Taxus baccata*. Docetaxel is approved for the treatment of breast and lung cancer, hormone-refractive prostate cancer and advanced gastric cancer. The antitumor mechanism of action for docetaxel is the hyperstabilisation of microtubules [23]. DCX binds preferentially to the tubulin β-subunit in the microtubules and promotes assembly of tubulin into stable microtubules while simultaneously inhibiting their depolymerisation. The formation of stable microtubule bundles disrupts the normal dynamic equilibrium between polymerization and depolymerisation within the microtubule system and leads to cell cycle arrest at the G2/M phase and, ultimately, cell death. Although this antitumor mechanism of action is similar to that of paclitaxel, DCX shows a higher affinity for the microtubule binding site and is approximately twice as potent as paclitaxel [24].

Although DCX showed significant antitumor activity against a broad spectrum of human tumours, it has very low water-solubility (6–7 µg/mL), requiring a formulation with surfactants such as polysorbate 80 or solvents such as ethanol to improve solubility [25]. Both polysorbate 80, a low molecular weight nonionic surfactant, and ethanol, have been reported to produce side-effects, including acute hypersensitivity reactions as well as pleural or pericardial effusions [26]. Therefore, the production of docetaxel-loaded nanoparticles is seen as a strategy to overcome the traditional formulation side effects and to improve drug efficacy through accumulation of the nanocarrier in the tumor parenchyma as a result of the enhanced permeation retention effect after parenteral administration [27]. Furthermore, such docetaxel nanocarriers could be used in novel loco-regional chemotherapeutic approaches, by inclusion either in implants for various solid tumors (potentially in combination with surgical tumor resection) [28,29] or in inhaled formulations for the lung tumors [30,31]. 

## 2. Materials and Methods 

### 2.1. Materials

Polyhydroxyalkanoates with three different comonomer compositions were synthesized in house using wild-type and transformant strains of *Cupriavidus malaysiensis* USMAA1020 isolated from Lake Kulim (Kulim, Malaysia) through a two-stage cultivation in shake-flasks, as it leads in higher 4HB molar fraction described elsewhere [22]. The three copolymer compositions used in this study were: 16 mol% (PHB16, Mw 282 ± 10 kDa), 30 mol% (PHB30, Mw 106 ± 4 kDa), and 70 mol% (PHB70, Mw 34 ± 2 kDa) of 4HB over 3HB ratio [32]. During the pre-formulation study, the surfactants used for nanoparticle production were polyvinyl alcohol (PVA, Mw10,000 Da, Sigma-Aldrich, Steinheim, Germany), polyoxyethylene-polyoxpropylene poloxamer block copolymer (Pluronic F-68, Sigma Aldrich, St. Louis, MO, USA), L-α-phosphatidylcholine from soybean Type II-S (Sigma-Aldrich, Steinheim, Germany) and vitamin E derivative D-α-tocopherol polyethylene glycol 1000 succinate (TPGS, International Laboratory, South San Francisco, CA, USA). The active ingredient, docetaxel (99+%) was obtained from International Laboratory (South San Francisco, CA, USA). Other solvents and chemicals such as dichloromethane, acetonitrile, sodium dihydrogen phosphate anhydrous, hydrochloric acid were of analytical grade.

### 2.2. Optimisation of P(3HB-co-4HB) Nanoparticles Production Conditions

The copolymer PHB70, i.e., P(3HB-*co*-70%4HB), was selected for the preliminary screening in terms of type and concentration of tensioactives to be used in nanoparticles preparation. P(3HB-co-4HB) nanoparticles were prepared using an oil-in-water (o/w) emulsion/solvent evaporation method slightly modified from Poletto and coworkers [33]. Briefly, P(3HB-*co*-4HB) (50.0 mg) was dissolved in 10.0 mL of dichloromethane. The aqueous phase (40.0 mL) contained 1.0% w/v PVA and different concentrations (0.03–0.5% w/v) of one tensioactive among lecithin, Pluronic F-68 and vitamin E TPGS. The organic solution was emulsified into the aqueous phase using a mechanical disperser for 5.0 min at 17,600 rpm (T25 digital Ultra-turrax, IKA, Staufen, Germany) and 5.0 min sonication using an ultrasound homogenizer (Labsonic M, Sartorius, Göttingen, Germany; operating frequency 30 kHz, output 100 W, 90% amplitude). The organic phase of the resulting emulsion was then evaporated overnight at room temperature under constant magnetic stirring at 400 rpm (RH digital, IKA, Staufen, Germany). 

Finally, PHB16, PHB30 and PHB70 nanoparticles were prepared as described above using an aqueous phase (40.0 mL) containing 1.0% w/v PVA and 0.03% w/v TPGS was added drop wise into the organic phase. Docetaxel loaded nanoparticles (DCX-PHB16, DCX-PHB30 and DCX-PHB70) were obtained by dissolving the drug in the organic phase along with the polymer. Nanoparticles with different drug/polymer percentage ratio by weight (5, 10, 20, 30, 40% w/w) were produced in order to evaluate drug loading capacity with all three copolymers.

All experiments were performed in triplicate.

### 2.3. P(3HB-co-4HB) Nanoparticles Physicochemical Characterization

The mean particle size, polydispersity index (PDI), and zeta potential were determined by dynamic light scattering using a ZetaSizer Nano-ZS 90 (Malvern Panalytical, Malvern, UK). The determination of nanoparticle sizes and PDI were carried out after dilution (1:100) in deionized water, at 25 °C. The measurement parameters used were as follows: material RI 1.59, material absorption 0.01, dispersant RI 1.330 and viscosity 0.8872. A total analysis time of 10 minutes per sample was used. 

The obtained suspension was determined for particle size and polydispersity while for zeta potential measurement, the same measurement process as described above were used. However, the operating parameters were as follows: dispersant RI 1.330, viscosity 0.8871 and dielectric constant 78.5, temperature 25 °C and number of runs 12. Each measurement was performed in triplicate. 

The shape and surface morphology of the produced nanoparticles were investigated by scanning electron microscopy (SEM) (Leo Supra 50VP Field Emission SEM, Carl-Ziess SMT, Oberkochen, Germany). SEM required a previous coating of the sample with gold, which was performed in a gold coating machine (SC515, Polaron Equipment Ltd., Watford, UK). Prior to viewing, samples were diluted (1:5) and dried at room temperature overnight before they were fixed on a double-sided sticky tape that was stuck to a standard sample stand.

### 2.4. Determination of Docetaxel-loaded P(3HB-co-4HB) Nanoparticles Encapsulation Efficiency 

The docetaxel was analyzed using a HPLC−UV method adapted from literature [34]. The HPLC system used was an Agilent 1260 Infinity LC System (Agilent Technologies Inc., Santa Clara, CA, USA) equipped with a UV detector set at 230 nm. The mobile phase was a 50:50 mixture of acetonitrile and phosphate buffer (0.02 M, pH 2) eluted with a flow rate of 0.5 mL/min. The analysis was carried out using a reverse-phase C18 column (Gemini, 3 × 150 mm, 5 µm, Phenomenex, Chromos, Singapore) maintained at 30 °C. Sample injection volume was 100 µL. Under these experimental conditions the total run time was 10 min and docetaxel (DCX) retention time was 6.7 min. The calibration curve was linear over the range of 0.05–20.00 µg/mL (*r^2^* > 0.9999). Limit of detection (LOD) of the method was calculated to be 29.63 ng/mL while limit of quantification (LOQ) was 89.80 ng/mL.

In order to characterize nanoparticles for encapsulation efficiency, nanoparticles were separated from larger particles and from the dispersant liquid by subsequent centrifugation steps. Initially, 10.0 mL of the nanoparticle suspension were centrifuged for 5.0 min at 5000 rpm (Sigma 318-K Benchtop centrifuge, Sortorius, Göttingen, Germany) to separate eventual agglomerates or precipitated drug from the nanoparticle suspension. The nanoparticle suspension was removed and the pellet obtained was dissolved in 5.0 mL acetonitrile and sonicated for 30.0 min before adding 5.0 mL of phosphate buffer (0.02 M, pH2) to proceed with HPLC quantification. To separate the free dissolved docetaxel in the suspension from the nanoparticles, 4.9 mL of the nanoparticle suspension was then ultracentrifuged for 1 h at 52,600 rpm (Optima Max-XP Ultracentrifuge, Beckman Coulter Inc., Calsbad, CA, USA). The recovered supernatant was directly analyzed with HPLC while the obtained pellet was resuspended in 2.0 mL dichloromethane and sonicated for 1 h with ultrasonic bath (FBI5055, Fisher Scientific, Waltham, MA, USA) to dissolve docetaxel loaded into nanoparticles. The dichloromethane solution was added to 10.0 mL of HPLC mobile phase and the chlorinated solvent was evaporated overnight at room temperature under magnetic stirring at 100 rpm before quantifying by HPLC analysis the encapsulated docetaxel. 

For analysis, the samples were solubilized with acetonitrile (1/5, *v*/*v*). Then the samples were transferred into auto-sampler vials, capped and placed in an HPLC auto-sampler. All samples were filtrated on 0.22 µm filters prior to injection into the HPLC apparatus. Then, 100 µL aliquots were injected into the HPLC column. Each sample was analyzed in triplicate. Drug loading was expressed as the amount of docetaxel (in µg) per mg of nanoparticles. 

### 2.5. In Vitro Drug Release Experiments

In vitro release studies of docetaxel from docetaxel loaded P(3HB-*co*-4HB) NPs were carried out using a dialysis membrane method reported in literature with some modifications [35]. Briefly, a volume of DCX-loaded P(3HB-*co*-4HB) NPs sufficient to reach a concentration of 500 µg/mL of total drug was put into a regenerated cellulose dialysis tube (Spectrum Spectra/Por 6, MW cut-off 12,000–14,000 Da, Fisher Scientific, Waltham, MA, USA) containing 8.0 mL total volume. Then, the dialysis bag was placed in 400.0 mL phosphate buffer solution (PBS, pH 7.4) to maintain sink conditions and stirred in an incubator shaker (Certomat IS, Sartorius, Göttingen, Germany) at 100 rpm and 37 °C. At regular time intervals, 1.0 mL of the release medium was removed and replaced with fresh PBS. The docetaxel released was quantified by HPLC. A control experiment to determine the release behavior of the free drug across the dialysis membrane was also performed placing the same amount of docetaxel as it was in DCX-P(3HB-*co*-4HB) NPs.

## 3. Results

### 3.1. Optimisation of P(3HB-co-4HB) Nanoparticle Production Conditions

**Figure 1:**
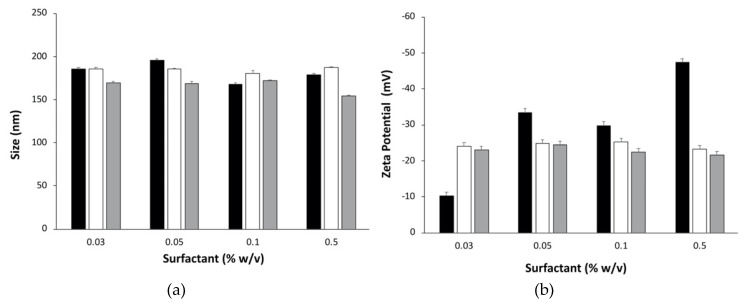
(**a**) Particle size and (**b**) zeta potential of PHB70 nanoparticles produced by emulsion/solvent evaporation method with 1.0% w/v PVA (Mw 10,000 Da) combined with different concentrations of tensioactives: lecithin (black bars), Pluronic F-68 (white bars) and TPGS (grey bars). Each value represents the mean and standard deviation of three independent replicates (*n* = 3).

Three surfactants, i.e., lecithin, Pluronic F-68 and TPGS, were chosen with the aim to investigate which type of tensioactive could work best for the production of P(3HB-co-4HB) nanoparticles by the emulsification/ solvent evaporation method. In this phase the copolymer PHB70 in combination with low molecular weight PVA as stabilizer in the aqueous phase. Figure 1 shows the mean particle size and PDI of the nanoparticles obtained by adding increasing concentrations (from 0.03% to 0.5% w/v) of each of the three surfactants in combination with 1.0% PVA. 

In general, the higher the concentration of the tensioactive, the smaller the size of the nanoparticles, however for the PHB70 nanoparticles obtained, it was found to be more or less the same size (below 200 nm) regardless of the tensioactive type and concentration (Figure 1a). Lecithin at 0.05% w/v provided the larger particles (195.2 nm) while the presence of TPGS as surfactant led to the smaller group of nanoparticles (150.0–172.0 nm). All PHB70 nanoparticles obtained showed with narrow size distribution with PDI in the range between 0.211 and 0.100. In particular, Pluronic F-68 provided the particles with the lowest PDI (all values lower than 0.125). Concerning the nanoparticle surface charge (Figure 1b), in the case of Pluronic F-68 and TGPS stabilized nanoparticles, the zeta potential values were negative (from −21.7 to −25.3 mV) and quite reproducible. When lecithin was used, the zeta potential values were still negative but extremely variable ranging from −10.3 mV to −47.4 mV. This variability could explain the fluctuating values evidenced both for size and PDI for lecithin-stabilized PHB70 nanoparticles. 

Taking into consideration all the data, it was considered that nanoparticles produced using 0.03% TPGS combined with 1.0% PVA 10,000 Da provided the best compromise in terms of PHB70 nanoparticles physicochemical characteristics: small particle size (169.6 nm), highly monodispersed (PDI 0.14) and acceptable surface charge value (−23.0 mV). 

Thus, considering these results, the manufacturing conditions including 0.03% TPGS with 1.0% PVA was chosen to be applied for the screening P(3HB-*co*-4HB) copolymer nanoparticle formulations. This choice was corroborated by a three-month stability study of unloaded P(3HB-*co*-4HB) that was conducted to investigate the storage conditions effect on P(3HB-*co*-4HB) nanoparticles physicochemical properties such as size, PDI and surface charge at 25 °C (room temperature), 4 °C (refrigerator) and 35 °C (see Supplementary Materials). All the formulations showed no marked differences in terms of physicochemical properties and no sign of precipitation or agglomeration (Figure S1). In fact, although average particle size slightly increased with respect to the initial values, all the nanoparticles’ mean particle size was still below 200 nm with low PDI (<0.2). These results indicate that P(3HB-*co*-4HB) nanoparticles manufactured in the selected conditions were stable and that the nanoparticles could be stored up to 90 days in different temperature conditions from 4 °C to 35 °C without major effects on their physicochemical characteristics.

### 3.2. Characterization of P(3HB-co-4HB) Nanoparticles with Different Incorporated Drug Concentration

Docetaxel-loaded P(3HB-*co*-4HB) nanoparticles were produced using copolymers characterized by different 4HB monomer contents, i.e., 16 mol% (PHB16), 30 mol% (PHB30) and 70 mol% (PHB70). The incorporation of the docetaxel drug into P(3HB-*co*-4HB) nanoparticles did not affect the size of the obtained nanoparticles when compared with blank P(3HB-co-4HB) nanoparticles Moreover, no significant particle size variation was evident for all three different 4HB monomer compositions of nanoparticles produced as shown in Table 1.

Blank and DCX-loaded PHB16, PHB30 and PNHB70 nanoparticles showed fairly constant particle size below 175.0 nm. No significant modification was evidenced for nanoparticles with a low drug loading (20% drug/polymer ration), while a slight increase in particle size was observed for the drug-loaded nanoparticles with a high drug loading (40% drug/polymer ratio). Moreover, a significant increase in PDI values for nanoparticles produced with PHB16 and PHB30 copolymers, indicated a broadening of particle size distribution, while only a slight increase in the polydispersity index was evidenced for PHB70-based nanoparticles loaded with docetaxel. Interestingly, the encapsulation of the drug in P(3HB-*co*-4HB) nanoparticles appeared to decrease the surface charge of the nanoparticles from around -20 mV to values below -10 mV. A significant increase in PDI values for nanoparticles produced with PHB16 and PHB30 copolymers, indicating a broadening of particle size distribution, while only a slight increase in polydispersity index was evidenced for PHB70-based nanoparticles loaded with docetaxel.

**Figure 2:**
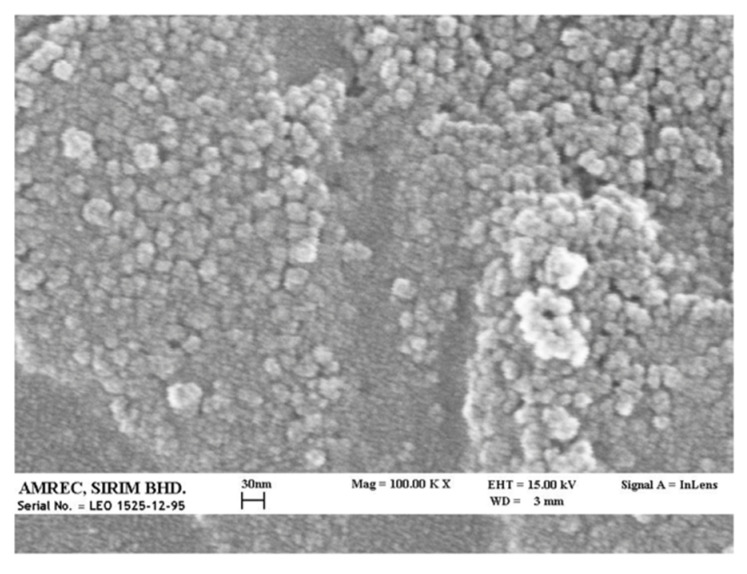
SEM image of DCX-loaded P(3HB-*co*-4HB) nanoparticles (DCX-PHB70).

Figure 2 shows a representative SEM image of P(3HB-*co*-4HB) nanoparticles prepared by solvent evaporation. The DCX-loaded P(3HB-*co*-4HB) nanoparticles were found to be spherical in shape even if with an irregular surface. The size of particles was quite homogeneous with dimensions well below 100 nm, while no drug crystals were observed. 

### 3.3. P(3HB-co-4HB) Nanoparticles Docetaxel Loading and Encapsulation Efficiency

The encapsulation efficiency of nanoparticles depends on several factors, including the physicochemical characteristics of the drug and core-forming polymer, the loading method and parameters [36].

Molecular weight, hydrophobic properties, 4HB monomer compositions as well as other parameters, such as the nature of the solvent used in the preparation method and DCX amount (5 to 40% drug/polymer ratio), were found to significantly impact DCX-loading efficiency into P(3HB-co-4HB) nanoparticles. 

**Figure 3:**
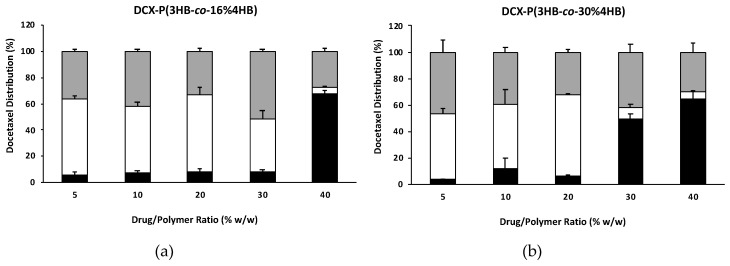

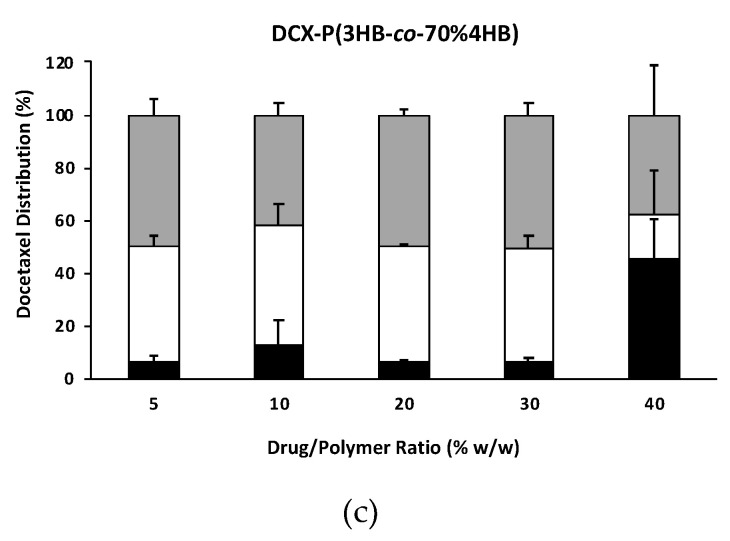
Docetaxel distribution, expressed as percentage of drug precipitated or in agglomerates (black bars), in solution (white bars) and encapsulated in nanoparticles (grey bars) in relation to the total drug recovered, determined for (**a**) DCX-PHB16, (**b**) DCX-PHB30 and (**c**) DCX-PHB70 nanoparticles.

Figure 3 shows that for all three copolymers tested encapsulation efficiencies ranged from 32 to over 51%. Drug precipitation was evident only for nanoparticles prepared with 40% drug/polymer using PHB16 (Figure 3a) and PHB70 copolymers (Figure 3c), while it was already evident at a 30% drug/polymer ratio for PHB30-based nanoparticles (Figure 3b). In the case of DCX-PHB16 and DCX-PHB30, encapsulation efficiencies appeared to be more variable with an average 40% value. On the other hand, DCX-PHB70 nanoparticles showed more reproducible encapsulation efficiency values quite close to 50% (Figure 3c). Furthermore, even if agglomerates were for the highest drug/polymer ratio, this did not cause an abrupt decrease in NP encapsulation efficiency as evidenced for the other copolymers. 

### 3.4. In vitro P(3HB-co-4HB) Nanoparticles Docetaxel Release

**Figure 4:**
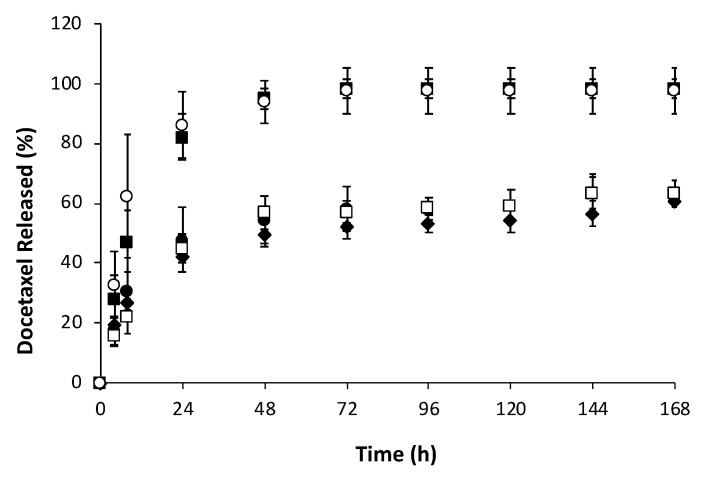
Docetaxel release profile from docetaxel-loaded PHB70 nanoparticles produced with different drug/polymer ratios: 5 (●), 10 (◆), 20 (□), 30 (■) and 40% (○) (*n* = 3, mean ± SD).

Drug release was studied for DCX-PHB70 produced at different drug/polymer ratios. In fact, overall, DCX-PHB70 nanoparticles demonstrated the most reliable encapsulation efficiency along with small particle size (~175.0 nm), negative surface charge (around −15 mV) and narrow particle size distribution (PDI <0.15). Drug release profiles are shown in Figure 4.

From the release profiles, two different behaviors exhibited by docetaxel-loaded PHB70 nanoparticles can be observed. P(3HB-co-4HB) nanoparticles with high drug/polymer ratio led to a relatively rapid release, while nanoparticles with drug/polymer ratio equal to or lower than 20% showed more controlled drug-release kinetics. In particular, nanoparticles with a drug/polymer ratio of 30 and 40% released more than 80% of docetaxel after 24 h and after 48 h the release was almost complete. In the case of lower drug/polymer ratios a biphasic release could be evidenced with an initial “burst” release of ~20–30% of docetaxel in the initial 8 h, followed by slower release rates with around 60% of drug released after 7 days. 

## 4. Discussion

Despite the growing interest in PHA applications in the biomedical field, most researchers have focused on their use as resorbable tissue-engineering materials, implants, tablets and microparticulate carriers, with a relatively low number of groups exploring their potential as nanoparticulate drug delivery systems [37]. For this reason, in this paper is presented a thorough investigation of the crucial parameters that could influence the production of P(3HB-co-4HB) nanoparticles. To start with, the screening of different types of tensioactives to be used in nanoparticle preparation by oil-in-water emulsion/solvent evaporation technique was carried out. The tensioactives selected, i.e., lecithin, Pluronic F-68 and vitamin E-derivative TPGS, were combined with PVA, which has been widely used in literature as a stabilizer not only for the production of polymer nanoparticles but also for PHB-based microparticles [38] and nanoparticles [39]. Lecithin, Pluronic F-68 and TPGS were investigated to determine whether these tensioactives could also produce the desired nanoparticles starting from P(3HB-co-4HB) copolymers. Lecithin was chosen since it is composed of phospholipids, natural components of cell membranes and regularly consumed as part of a normal diet. It is used extensively in pharmaceutical applications as an emulsifying, dispersing, and stabilizing agent and is included in intramuscular and intravenous injectables and other parenteral nutrition formulation [40]. Lecithin is a complex mixture of phosphatides consisting of phosphatidylcholine, phosphatidylethanolamine, phosphatidylserine, phosphatidylinositol and other substances such as triglycerides and fatty acids. It is approved by the FDA and included in the 2013 Inactive Ingredients Guide for Parenterals (e.g., 0.3–2.3% for intramuscular injection). Pluronic F68 is a poloxamer, i.e., a triblock copolymer based on ethylene and propylene oxides. Poloxamers are used as antifoaming agents, wetting agents, dispersants, thickeners, and emulsifiers. Pluronic F68 is mainly used as a nonionic surfactant and is relatively nontoxic. Due to their amphiphilic character, poloxamers can stabilize nanoparticles’ surfaces and mediate the interaction between the polymer and the drug, thus avoiding the formation of flakes and precipitates [41]. Alpha-tocopherol derivative TPGS is a water-soluble form of vitamin E and a polyoxyethylated surfactant [42]. Because of the potential additional adjuvant effects provided by vitamin E antioxidant properties in several diseases, TPGS has been proposed as surfactant in a number of different nanoformulations (micelles, nanoemulsions, solid lipid nanoparticles, etc.) for several administration routes, such as oral [43,44], topical [45], nasal [46], pulmonary [47] and ocular delivery [48]. The screening results evidenced that the best surfactant for P(3HB-co-4HB) nanoparticle production by the emulsification/solvent evaporation method was TPGS in association with PVA since this allowed for the production of stable monodisperse nanoparticles with particle size around 170 nm and negative surface charge. Pluronic stabilized nanoparticles showed a slightly larger particle size, while lecithin led to more variable results in terms of surface charge. These differences could be attributed to the polymeric nature of Pluronic surfactant in one case and to the variability of the soybean-derived lecithin mixture in the latter.

When different P(3HB-*co*-4HB) copolymers (PHB16, PHB30 and PHB70) were used for nanoparticle production and docetaxel was encapsulated at various drug concentrations, generally the sizes of all the nanoparticles produced did not exceed the 200 nm size which indicated that the nanoparticle size was not affected by the 4HB monomer content in the copolymer or by the drug loading ratio. The electron microscopy images of docetaxel-loaded P(3HB-*co*-4HB) nanoparticles evidenced particles with an irregular shape and corrugated surface but quite uniformly distributed and the absence of evident drug crystals. The average size of the nanoparticles observed in the SEM images was smaller than that determined using dynamic light scattering. However, this is often observed since the particle size determined by DLS measures the hydrodynamic diameter, i.e., takes into account also the aqueous layer that surrounds the particles. In the case of SEM, the sample is dried and metal coated, this leads to the collapse of the hydration layer, reducing significantly the apparent size of the nanoparticles observed [49]. Furthermore, drug loading results suggested that P(3HB-*co*-4HB) nanoparticles produced with the copolymer with the highest 4HB monomer content (PHB70) was able to incorporate the anticancer drug docetaxel with better reproducibility. In fact, even if DCX-loaded P(3HB-*co*-4HB) nanoparticles could be prepared with all three different copolymers without affecting nanoparticle average particle size, lower polydispersity index values were obtained for nanoparticles produced using PHB70. Concerning the nanoparticle surface charge, blank nanoparticles showed negative surface charge, as expected due to the presence of terminal carboxylic groups in the P(3HB-*co*-4HB) polymer chains. However, when docetaxel was loaded in P(3HB-*co*-4HB) nanoparticles a decrease in zeta potential value was evident. This result appears to be due to the presence of drug molecules at the surface of nanoparticles produced being able to mask the negative charge-bearing polymer chains, as already evidenced by Musumeci and colleagues in the case of docetaxel-loaded PLA nanoparticles [50]. Moreover, the increased proportion of agglomerates and/or precipitated drug shown by nanoparticles at high drug content could be explained, at least in part, by the reduced electrostatic stabilization of nanoparticles. This may be due to the fact that an excess of DCX in the formulation leads to a higher proportion of DCX molecules in the nanoparticle/liquid instead of encapsulated in the nanoparticle polymer core during the preparation stage. This eventually can induce precipitation of the drug and/or the formation of nanoparticle agglomerates, as shown by the abrupt reduction in the nanoparticle encapsulation efficiencies at high drug/polymer ratios. In any case, the encapsulation efficiencies obtained, with values close to 50% for nanoparticles prepared using a PBH70 copolymer, appear very promising, considering that docetaxel encapsulation efficiencies for PLA and PLGA nanoparticles were reported to be well below 25% using just 1% drug/polymer ratio [50].

Docetaxel release appears to be dependent on drug/polymer ratio. Drug release from nanoparticulate systems is governed by several factors, including drug location inside the vector and the ease with which the drug molecules are able to diffuse through the nanoparticle core matrix. In particular, burst effects are typically attributed to the release of drug molecules located near the particle surface, upon exposure to sink conditions. In our case, two release patterns were evidenced with faster release (complete release within 48 h) evidenced by nanoparticles producing a high drug/polymer ratio and a burst followed by a prolonged release (60% released after 7 days) for drug/polymer ratios lower than 30%. Interestingly, the group of Leroux comparing polyester nanoparticles loaded with paclitaxel, reported that 90% of the cargo was immediately released in the case of polyhydroxyalkanoate (PHA) nanoparticles, while obtaining a bimodal release for nanoparticles prepared using PLA and PLGA, with a burst release followed by sustained release up to 5 days, mainly dependent on polymer composition. In particular, the authors attribute the poor encapsulation and fast release of paclitaxel from PHA nanoparticles to a poor drug−polymer compatibility [35]. In our case however, the release profiles are consistent with an initial rapid release of the fraction of the drug more superficially linked to the nanoparticles followed by a controlled release of the drug encapsulated in the polymer matrix [51]. The prolonged release might be due not only to drug diffusion but also to the erosion and degradation of the polymer matrix, since DCX has very poor solubility in water [52]. These results clearly evidence that, in terms of potential drug delivery applications, PHAs, and in particular the naturally derived P(3HB-*co*-4HB) copolymer, show similar, if not superior, features to widely used biomedical polymers, such as PLA and PLGA.

## 5. Conclusions

The results of the present research have demonstrated that it was possible to prepare nanoparticles using the biodegradable polyester P(3HB-*co*-4HB) obtained in-house from *Cupriavidus malaysiensis* USMAA1020 using an emulsion/solvent evaporation method. Furthermore, P(3HB-*co*-4HB) nanoparticles were loaded with the anticancer docetaxel, selected as a model drug. Docetaxel, an anticancer drug with poor aqueous solubility, was successfully encapsulated into nanoparticles without affecting the nanosystem physicochemical properties. The study showed that the release profile was influenced by the drug/polymer ratio. The nanoparticles produced showed an average particle size of 140–180 nm, PDI value of 0.09 and the particles had smooth surfaces, spherical in shape and no free drug crystals attached to the particles. Results showed that P(3HB-*co*-70%4HB) polymer can load docetaxel inside the nanoparticles with no aggregation up to a drug/polymer ratio of 30% with encapsulation efficiency ~50%. Furthermore, the release kinetics study also showed that low drug/polymer ratios could lead to a prolonged release typical of other polyester carriers such as PLGA. High drug loading however led to a more rapid release rate with almost the entire encapsulated drug released within 48 h. These properties showed that P(3HB-co-70%4HB) nanoparticles can be a prospective drug carrier candidate for a controlled release application.

## Figures and Tables

**Table 1:** Characteristics of blank and docetaxel (DCX)-loaded P(3HB-*co*-4HB) nanoparticles prepared by O/W emulsion solvent evaporation method using copolymers with different 4HB monomer content (mean ± SD, *n* = 3).

	Nanoparticles Characteristics				
	Size (nm)	PDI	Zeta Potential (mV)	Drug/Polymer Ratio (% w/w)	DCX Conc. (mg/ml)
**PHB16**	168.2 ± 0.9	0.121 ± 0.002	−15.5 ± 2.2	-	-
**PHB30**	174.4 ± 1.5	0.138 ± 0.005	−21.0 ± 0.8	-	-
**PHB70**	169.3 ± 1.1	0.112 ± 0.012	−20.5 ± 1.2	-	-
**DCX- PHB16**	153.4 ± 1.4	0.129 ± 0.015	−11.4 ± 3.5	20	0.5
**DCX- PHB30**	145.8 ± 0.7	0.099 ± 0.016	−15.9 ± 1.6	20	0.5
**DCX- PHB70**	147.1 ± 1.2	0.095 ± 0.019	−14.7 ± 1.4	20	0.5
**DCX- PHB16**	206.0 ± 4.5	0.347 ± 0.020	−9.7 ± 1.5	40	1.0
**DCX- PHB30**	192.3 ± 2.9	0.370 ± 0.020	−8.1 ± 0.8	40	1.0
**DCX- PHB70**	183.9 ± 1.3	0.169 ± 0.008	−9.3 ± 0. 7	40	1.0

## Supplementary Materials

The following are available online at https://www.mdpi.com/2079-4991/10/11/2123/s1, Figure S1: Particle size (bars) and PDI values (lines) of P(3HB-co-4HB) nanoparticles stored at different temperature for 90 days: (a) PHB16; (b) PHB30 and (c) PHB70.

## References

[1] George A., Shah P.A., Shrivastav P.S. (2019). Natural biodegradable polymers based nano-formulations for drug delivery: A review. Int. J. Pharm..

[2] Zhao K., Li D., Shi C., Ma X., Rong G., Kang H., Wang X., Sun B. (2016). Biodegradable Polymeric Nanoparticles as the Delivery Carrier for Drug. Curr. Drug Deliv..

[3] Soppimath K.S., Aminabhavi T.M., Kulkarni A.R., Rudzinski E.W. (2001). Biodegradable polymeric nanoparticles as drug delivery devices. J. Control. Release.

[4] De Haan B.J., Rossi A., Faas M.M., Smelt M.J., Sonvico F., Colombo P., De Vos P. (2011). Structural surface changes and inflammatory responses against alginate-based microcapsules after exposure to human peritoneal fluid. J. Biomed. Mater. Res. Part. A.

[5] Gerelli Y., Di Bari M.T., Deriu A., Cantu L., Colombo P., Como C., Motta S., Sonvico F., May R. (2008). Structure and organization of phospholipid/polysaccharide nanoparticles. J. Physics Condens. Matter.

[6] Martin D.P., Williams S.F. (2003). Medical applications of poly-4-hydroxybutyrate: A strong flexible absorbable biomaterial. Biochem. Eng. J..

[7] Barouti G., Jaffredo C.G., Guillaume S.M. (2017). Advances in drug delivery systems based on synthetic poly(hydroxybutyrate) (co)polymers. Prog. Polym. Sci..

[8] Sudesh K., Abe H., Doi Y. (2000). Synthesis, structure and properties of polyhydroxyalkanoates: Biological polyesters. Prog. Polym. Sci..

[9] Saito Y., Nakamura S., Hiramitsu M., Doi Y. (1996). Microbial synthesis and properties of poly(3-hydroxybutyrate-co-4-hydroxybutyrate). Polym. Int..

[10] Chee J.-W., Amirul A., Muhammad T.T., Majid M., Mansor S. (2008). The influence of copolymer ratio and drug loading level on the biocompatibility of P(3HB-co-4HB) synthesized by Cupriavidus sp. (USMAA2-4). Biochem. Eng. J..

[11] Valentin E.H., Dennis D. (1997). Production of poly(3-hydroxybutyrate-co-4-hydroxybutyrate) in recombinant Escherichia coli grown on glucose. J. Biotechnol..

[12] Mukai K., Yamada K., Doi Y. (1994). Efficient hydrolysis of polyhydroxyalkanoates by Pseudomonas stutzeri YM1414 isolated from lake water. Polym. Degrad. Stab..

[13] Saito Y., Doi Y. (1994). Microbial synthesis and properties of poly(3-hydroxybutyrate-co-4-hydroxybutyrate) in Comamonas acidovorans. Int. J. Biol. Macromol..

[14] Ying T.H., Ishii D., Mahara A., Murakami S., Yamaoka T., Sudesh K., Samian R., Fujita M., Maeda M., Iwata T. (2008). Scaffolds from electrospun polyhydroxyalkanoate copolymers: Fabrication, characterization, bioabsorption and tissue response. Biomaterials.

[15] Xu X.-Y., Li X.-T., Peng S.-W., Xiao J.-F., Liu C., Fang G., Chen K.C., Chen G.-Q. (2010). The behaviour of neural stem cells on polyhydroxyalkanoate nanofiber scaffolds. Biomaterials.

[16] Rao U., Sridhar R., Sehgal P. (2010). Biosynthesis and biocompatibility of poly(3-hydroxybutyrate-co-4-hydroxybutyrate) produced by Cupriavidus necator from spent palm oil. Biochem. Eng. J..

[17] Shrivastav A., Kim H.-Y., Kim Y.-R. (2013). Advances in the Applications of Polyhydroxyalkanoate Nanoparticles for Novel Drug Delivery System. BioMed Res. Int..

[18] Nigmatullin R., Thomas P., Lukasiewicz B., Puthussery H., Roy I. (2015). Polyhydroxyalkanoates, a family of natural polymers, and their applications in drug delivery. J. Chem. Technol. Biotechnol..

[19] Pignatello R., Musumeci T., Impallomeni G., Carnemolla G.M., Puglisi G., Ballistreri A. (2009). Poly(3-hydroxybutyrate-co-ε-caprolactone) copolymers and poly(3-hydroxybutyrate-co-3-hydroxyvalerate-co-ε-caprolactone) terpolymers as novel materials for colloidal drug delivery systems. Eur. J. Pharm. Sci..

[20] O’Connor S., Szwej E., Nikodinovic J., O’Connor A., Byrne A.T., Devocelle M., O’Donovan N., Gallagher W.M., Babu R., Kenny S.T. (2013). The anti-cancer activity of a cationic anti-microbial peptide derived from monomers of polyhydroxyalkanoate. Biomaterials.

[21] Ali I., Jamil N. (2016). Polyhydroxyalkanoates: Current applications in the medical field. Front. Biol..

[22] Vigneswari S., Vijaya S., Majid M.I.A., Sudesh K., Sipaut C.S., Azizan M.N.M., Amirul A.A. (2009). Enhanced production of poly(3-hydroxybutyrate-co-4-hydroxybutyrate) copolymer with manipulated variables and its properties. J. Ind. Microbiol. Biotechnol..

[23] Montero A., Fossella F., Hortobagyi G., Valero V. (2005). Docetaxel for treatment of solid tumours: A systematic review of clinical data. Lancet Oncol..

[24] Jordan M.A., Wilson L. (2004). Microtubules as a target for anticancer drugs. Nat. Rev. Cancer.

[25] Du W., Hong L., Yao T., Yang X., He Q., Yang B., Hu Y. (2007). Synthesis and evaluation of water-soluble docetaxel prodrugs-docetaxel esters of malic acid. Bioorganic Med. Chem..

[26] Engels F.K., Mathot R.A.A., Verweij J. (2007). Alternative drug formulations of docetaxel: A review. Anti-Cancer Drugs.

[27] Brigger I., Dubernet C., Couvreur P. (2002). Nanoparticles in cancer therapy and diagnosis. Adv. Drug Deliv. Rev..

[28] Ampollini L., Barocelli E., Cavazzoni A., Petronini P., Mucchino C., Cantoni A.M., Leonardi F., Ventura L., Barbieri S., Colombo P. (2018). Polymeric films loaded with cisplatin for malignant pleural mesothelioma: A pharmacokinetic study in an ovine model. J. Thorac. Dis..

[29] Sonvico F., Barbieri S., Colombo P., Barocelli E., Mucchino C., Cantoni A.M., Petronini P.G., Rusca M., Carbognani P., Ampollini L. (2018). Combined hyaluronate-based films loaded with pemetrexed and cisplatin for the treatment of malignant pleural mesothelioma: Preliminary evaluation in an orthotopic tumor recurrence model. Eur. J. Pharm. Sci..

[30] Pereira G.G., Lawson A.J., Buttini F., Sonvico F. (2015). Loco-regional administration of nanomedicines for the treatment of lung cancer. Drug Deliv..

[31] Gagnadoux F., Hureaux J., Vecellio L., Urban T., Le Pape A., Valo I., Montharu J., Leblond V., Boisdron-Celle M., Lerondel S. (2008). Aerosolized Chemotherapy. J. Aerosol Med. Pulm. Drug Deliv..

[32] Huong K.-H., Yahya A., Amirul A.A. (2013). Pronounced synergistic influence of mixed substrate cultivation on single step copolymer P(3HB-co-4HB) biosynthesis with a wide range of 4HB monomer composition. J. Chem. Technol. Biotechnol..

[33] Poletto F.S., Jäger E., I Ré M., Guterres S.S., Pohlmann A.R. (2007). Rate-modulating PHBHV/PCL microparticles containing weak acid model drugs. Int. J. Pharm..

[34] Agüeros M., Ruiz-Gatón L., Vauthier C., Bouchemal K., Espuelas S., Ponchel G., Irache J. (2009). Combined hydroxypropyl-β-cyclodextrin and poly(anhydride) nanoparticles improve the oral permeability of paclitaxel. Eur. J. Pharm. Sci..

[35] Song X., Zhao Y., Hou S., Xu F., Zhao R., He J., Cai Z., Li Y., Chen Q. (2008). Dual agents loaded PLGA nanoparticles: Systematic study of particle size and drug entrapment efficiency. Eur. J. Pharm. Biopharm..

[36] Gaucher G., Marchessault R.H., Leroux J.-C. (2010). Polyester-based micelles and nanoparticles for the parenteral delivery of taxanes. J. Control. Release.

[37] Williams S.F., Martin D.P. (2005). Applications of Polyhydroxyalkanoates (PHA) in Medicine and Pharmacy.

[38] Maia J.L., Santana M.H.A., Ré M.I. (2004). The effect of some processing conditions on the characteristics of biodegradable microspheres obtained by an emulsion solvent evaporation process. Braz. J. Chem. Eng..

[39] Nachiyar C.V., Devi A.B., Namasivayam S., Raja K., Rabel A.M. (2015). Levofloxacin Loaded Polyhydroxybutyrate Nanodrug Conjugate for In-Vitro Controlled Drug Release. Res. J. Pharm. Biol. Chem. Sci..

[40] Lixin W., Haibing H., Xing T., Ruiying S., Dawei C. (2006). A less irritant norcantharidin lipid microspheres: Formulation and drug distribution. Int. J. Pharm..

[41] Errico C., Bartoli C., Chiellini F., Chiellini E. (2009). Poly(hydroxyalkanoates)-Based Polymeric Nanoparticles for Drug Delivery. J. Biomed. Biotechnol..

[42] Jiao J. (2008). Polyoxyethylated nonionic surfactants and their applications in topical ocular drug delivery. Adv. Drug Deliv. Rev..

[43] Romana B., Hassan M., Sonvico F., Pereira G.G., Mason A.F., Thordarson P., Bremmell K.E., Barnes T.J., Prestidge C.A. (2020). A liposome-micelle-hybrid (LMH) oral delivery system for poorly water-soluble drugs: Enhancing solubilisation and intestinal transport. Eur. J. Pharm. Biopharm..

[44] Pereira G.G., Rawling T., Pozzoli M., Pazderka C., Chen Y., Dunstan C., Murray M., Sonvico F. (2018). Nanoemulsion-Enabled Oral Delivery of Novel Anticancer ω-3 Fatty Acid Derivatives. Nanomaterials.

[45] Telò I., Del Favero E., Cantù L., Frattini N., Pescina S., Padula C., Santi P., Sonvico F., Nicoli S. (2017). Gel-like TPGS-Based Microemulsions for Imiquimod Dermal Delivery: Role of Mesostructure on the Uptake and Distribution into the Skin. Mol. Pharm..

[46] Di Cola E., Cantu’ L., Brocca P., Rondelli V., Fadda G.C., Canelli E., Martelli P., Clementino A., Sonvico F., Bettini R. (2019). Novel O/W nanoemulsions for nasal administration: Structural hints in the selection of performing vehicles with enhanced mucopenetration. Colloids Surf. B Biointerfaces.

[47] Rossi I., Sonvico F., McConville J.T., Rossi F., Fröhlich E., Zellnitz S., Rossi A., Del Favero E., Bettini R., Buttini F. (2018). Nebulized coenzyme Q 10 nanosuspensions: A versatile approach for pulmonary antioxidant therapy. Eur. J. Pharm. Sci..

[48] Grimaudo M.A., Pescina S., Santi P., Santi P., Concheiro A., Alvarezlorenzo C., Nicoli S. (2018). Poloxamer 407/TPGS Mixed Micelles as Promising Carriers for Cyclosporine Ocular Delivery. Mol. Pharm..

[49] Bootz A., Vogel V., Schubert D., Kreuter J. (2004). Comparison of scanning electron microscopy, dynamic light scattering and analytical ultracentrifugation for the sizing of poly(butyl cyanoacrylate) nanoparticles. Eur. J. Pharm. Biopharm..

[50] Musumeci T., Ventura C., Giannone I., Ruozi B., Montenegro L., Pignatello R., Puglisi G. (2006). PLA/PLGA nanoparticles for sustained release of docetaxel. Int. J. Pharm..

[51] Ling Y., Wei K., Luo Y., Gao X., Zhong S. (2011). Dual docetaxel/superparamagnetic iron oxide loaded nanoparticles for both targeting magnetic resonance imaging and cancer therapy. Biomaterials.

[52] Yanasarn N., Sloat B.R., Cui Z. (2009). Nanoparticles engineered from lecithin-in-water emulsions as a potential delivery system for docetaxel. Int. J. Pharm..

