# Supplementary material for: Docetaxel-Loaded Poly(3HB-co-4HB) Biodegradable Nanoparticles: Impact of Copolymer Composition
Source: Nanomaterials (Basel). 2020 Oct 26;10(11):2123. doi: 10.3390/nano10112123 (PMC7716210; doi:10.3390/nano10112123)
Supplement: Supplementary file 1 [file nanomaterials-10-02123-s001.pdf]

nanomaterials

MDPI

Supplementary Materials

Docetaxel-Loaded Poly(3HB-*co*-4HB) Biodegradable Nanoparticles: Impact of Copolymer Composition

Ahmad Fisol Faisalina [1], Fabio Sonvico [2,*], Paolo Colombo [2], Al-Ashraf Abdullah Amirul [1,3], Habibah A. Wahab [1,*] and Mohamed Isa Abdul Majid [1]

1 Malaysian Institute of Pharmaceuticals and Nutraceuticals (IPharm), National Institute of Biotechnology Malaysia (NIBM), Ministry of Science, Technology and Innovation (MOSTI), Penang 11800, Malaysia; faisalinafisol@gmail.com (F.A.F.); amirul@usm.my (A.A.A); isa_majid@usm.my (M.I.A.M)

2 Food and Drug Department, University of Parma, 43124 (PR) Parma, Italy; paolo.colombo@unipr.it

3 School of Biological Sciences, Universiti Sains Malaysia, Penang 11800, Malaysia; amirul@usm.my (A.A.A)

* Correspondence: fabio.sonvico@unipr.it (F.S.); bibwahab@gmail.com (H.A.W.); Tel.: +39-0521906282 (F.S.); Tel.: +604-6577888 (Ext.2228) (H.A.W.)

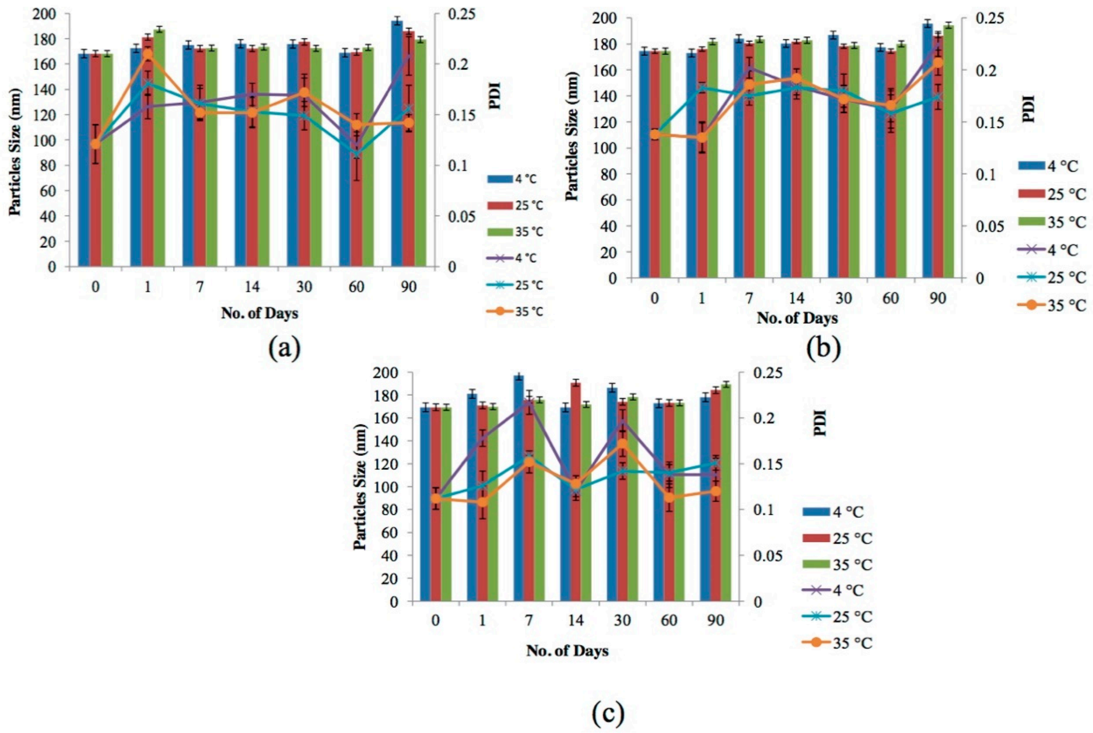

Figure S1. Particle size (bars) and PDI values (lines) of P(3HB-co-4HB) nanoparticles stored at different temperature for 90 days: (**a**) PHB16; (**b**) PHB30 and (**c**) PHB70.
